# Promising Targets and Current Clinical Trials in Metastatic Non-Squamous NSCLC

**DOI:** 10.3389/fonc.2014.00329

**Published:** 2014-11-25

**Authors:** Alona Zer, Natasha Leighl

**Affiliations:** ^1^Division of Medical Oncology, Princess Margaret Cancer Centre, University of Toronto, Toronto, ON, Canada

**Keywords:** NSCLC, nuclear targets, intracellular pathways, *EGFR*, *ALK*, novel targets, non-squamous

## Abstract

Lung adenocarcinoma is the most common subtype of lung cancer today. With the discovery of epidermal growth factor receptor (*EGFR*) mutations, anaplastic lymphoma kinase (*ALK*) rearrangements, and effective targeted therapy, personalized medicine has become a reality for patients with lung adenocarcinoma. Here, we review potential additional targets and novel therapies of interest in lung adenocarcinoma including targets within the cell surface (receptor tyrosine kinases *EGFR*, human epidermal growth factor receptor 2, *RET*, *ROS1*, mesenchymal–epidermal transition, TRK), targets in intracellular signal transduction (*ALK*, RAS–RAF–*MEK*, PI3K–*AKT*–PTEN, WNT), nuclear targets such as poly-ADP ribose polymerase, heat shock protein 90, and histone deacetylase, and selected pathways in the tumor environment. With the evolving ability to identify specific molecular aberrations in patient tumors in routine practice, our ability to further personalize therapy in lung adenocarcinoma is rapidly expanding.

## Introduction

In recent years, we have witnessed a transformation of the treatment paradigm for advanced non-small cell lung cancer (NSCLC). Previously, patients were offered platinum-based chemotherapy, followed by second-line chemotherapy with docetaxel or pemetrexed, and erlotinib after chemotherapy failure, yielding modest benefits in an unselected population (1). Using molecular selection, clinical trials of targeted therapy have demonstrated major improvements in response, quality of life, and progression-free survival compared to chemotherapy, using epidermal growth factor receptor (*EGFR*) TKI in *EGFR* mutant NSCLC and crizotinib in anaplastic lymphoma kinase (*ALK*) rearranged NSCLC (2, 3). Survival is similar in many of these trials, given the high rate of crossover from chemotherapy to the more active agent upon progression.

**Figure 1:**
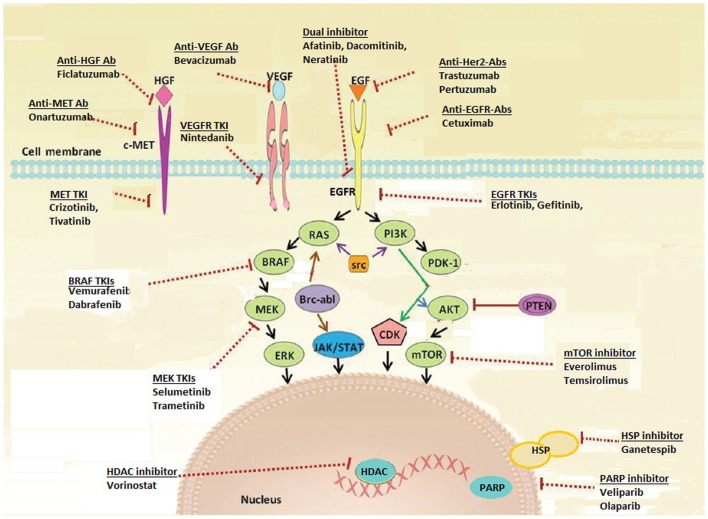
**Targetable pathways in the non-squamous NSCLC cell**.

It is now standard of care to test non-squamous lung carcinoma for the presence of *EGFR* mutation and *ALK* rearrangement upon diagnosis of advanced disease (4), in order to select patients for initial *EGFR* TKI and *ALK* inhibitor therapy. The remarkable activity of these agents in molecularly selected lung cancer patients has led to a rapid increase in studies evaluating new targets and novel targeted agents. These targets include oncogenic driver mutations (genomic alterations that initiate malignant transformation of the normal cell), signal transduction proteins, tumor angiogenesis, and factors in the tumor environment supporting cancer cell proliferation (for example, immune-modulating proteins) (Figure 1; Table 1). In this review, we discuss selected new and promising targets as well as targeted therapies currently under investigation in non-squamous NSCLC, specifically adenocarcinoma.

**Table 1:** **Selected targets and selected targeted agents in lung adenocarcinoma**.

Target	Frequency (common clinical features)	Selected agents under study	Current development
**CELL SURFACE TARGETS**			
*EGFR* mutations (*EGFR* TKI acquired resistance)	17–43% (female, never smokers, Asian)	AZD9291	Phase II/III
		CO-1686	
		HM-61713	
		Afatinib + cetuximab	
		Erlotinib + AUY922	
		Gefitinib + INC280 (*MET* TKI)	
*HER2* insertion mutation	3–6% (female, never smokers)	Trastuzumab + chemotherapy	Phase II
		Afatinib	
		Pertuzumab	
		Neratinib + temsirolimus	
*RET* fusion	1–2% (young, never smokers, poorly differentiated tumor)	Vandetanib	Phase II
		Cabozantinib	
		Lenvatinib	
		Ponatinib	
**INTRACELLULAR PATHWAYS**			
*ROS1* fusion	1–2% (young, never smokers)	Crizotinib	Phase I/II
		Ceritinib	
		AP26113	
		Foretinib	
		PF-06463922	
		AT13387	
*MET* amplification	~1%	Crizotinib	Phase I
*MET* expression	Up to 50%	Ficlatuzumab	Phase II/III
*NTRK-1* fusion	~1%	RXDX101	Phase I
*KRAS* mutations	Up to 30%	Selumetinib + chemotherapy	Phase I–III
		Trametinib + chemotherapy	
		MEK162 + chemotherapy	
*BRAF* mutation	3%, smokers	Dabrafenib	Phase I/II
		Vemurafenib	
mTOR activation	Up to 90%	Everolimus	Phase II
		Temsirolimus	
		Sirolimus	
**NUCLEAR TARGETS**			
PARP	n/a	Olaparib + chemotherapy	Phase II/III
		Veliparib + chemotherapy	
HDAC	n/a	Romidepsin	Phase II
		Pabinostat	
		Etinostat	
**TUMOR ENVIRONMENT**			
RANK-Ligand	n/a	Denosumab + chemotherapy	Phase III
VEGF	n/a	Bevacizumab	Phase II/III
		Nintedanib	
CTLA-4	n/a	Ipilimumab	Phase II/III
PD-1	~40% of lung adenocarcinomas express PD-L1	Nivolumab	Phase II/III
PD-L1		Lambrolizumab	
		BMS-936559	
		MPDL-3286A	

*n/a, not available; *EGFR* TKI, epidermal growth factor receptor tyrosine kinase inhibitor; NTRK, neurotrophic tyrosine kinase receptor type; mTOR, mammalian target of rapamycin; PARP, poly-ADP ribose polymerase; HDAC, histone deacetylases; RANKL, receptor activator of nuclear factor kappa-B ligand; CTLA-4, cytotoxic T-lymphocyte-associated protein 4; PD-1, programed cell death protein 1; PD-L1, programed cell death protein 1 ligand*.

## Targets within the Cell Surface

### Epidermal growth factor receptor

Targeting *EGFR* has led to a breakthrough in understanding of lung cancer biology, and the NSCLC treatment paradigm. Mutations in *EGFR*, resulting in greater affinity for ATP binding by the *EGFR* tyrosine kinase domain and constitutive activation, are found in ~15% of lung cancers in Caucasians and 40% in Asians (5, 6). Activating mutations are significantly associated with response to *EGFR* TKIs, with erlotinib, gefitinib, and afatinib established as initial standard therapy. However, resistance mutations have been identified, such as T790M in exon 20. There are multiple agents in development with enhanced affinity for T790M mutant lung cancer that may spare wild type *EGFR*, potentially avoiding toxicities like rash and diarrhea. AZD9291 and CO-1686 are examples of such agents, and have reported responses in 58–64% of patients with acquired *EGFR* TKI resistance and documented T790M mutation (7, 8). There are other strategies in development, targeting acquired *EGFR* TKI resistance including chemotherapy with intercalated *EGFR* TKI, combinations with mesenchymal–epidermal transition (*MET*), dual *EGFR* and heat shock protein 90 (HSP90) inhibitors, and more. For example, combination of afatinib and cetuximab has demonstrated activity in patients with acquired *EGFR* TKI resistance and T790M positive and negative tumors (9), and the addition of AUY922 to erlotinib has restored sensitivity in 22% of patients with acquired resistance to erlotinib (10).

### Human epidermal growth factor receptor 2

Human epidermal growth factor receptor 2 is a cell surface receptor, and member of the erbB receptor tyrosine kinase family. It is activated by heterodimerization with other ligand-bound members of the erbB family, or by homodimerization. *HER2* is a key oncogene in breast cancer, and is associated with improved outcomes with trastuzumab (anti-*HER2* monoclonal antibody) (11, 12). In NSCLC, *HER2* protein overexpression is found in 6–35% of patients and *HER2* gene amplification is found in 10–20% (13). Trastuzumab has shown minimal activity in lung cancer, both as a single agent and in combination with chemotherapy, particularly in patients with FISH positive or IHC 3+ tumors (14, 15).

*HER2* mutations are seen in 2.8–6% of lung adenocarcinomas (16, 17), more commonly in women and non-smokers. These mutations are commonly exon 20 in-frame insertions. Activity has been seen with trastuzumab-based therapy and afatinib (13, 18). A phase I trial of neratinib (an irreversible pan-HER inhibitor) and temsirolimus (mTOR inhibitor) suggested benefit in five patients with *HER2*-mutant NSCLC (19). A phase II trial assessing this combination is underway. Other trials include studies of *HER2*-directed antibodies (trastuzumab, pertuzumab), TKIs (neratinib, dacomitinib, and afatinib), and a peptide vaccine (www.clinicaltrials.gov).

### *RET* 

*RET* (rearranged during transfection), is a known oncogene in thyroid cancer, with both activating mutations and gene rearrangements observed (20). Approximately 1.5% of NSCLC cases have *RET* translocations, typically in younger, non-smoking adenocarcinoma patients (21). Fusion variants include *KIF5B-RET* in adenocarcinoma, CCDC6, NCO4, and TRIM33 also found in thyroid cancer (22, 23).

Vandetanib, sunitinib, sorafenib, lenvatinib, ponatinib, and cabozantinib are all multi-targeted kinase inhibitors that target *RET*. Activity has been seen in *RET*-positive lung cancer patients with cabozantinib and vandetinib, and multiple trials are ongoing in this population with a recent halt in a ponatinib study for safety concerns (24, 25).

### *ROS1* 

*ROS1* encodes a receptor tyrosine kinase of the insulin receptor super family, with no known ligand and little known about its normal function. *ROS1* fusion genes, with oncogenic transformation potential, have been described in multiple tumor cell lines, including lung cancer. The prevalence of *ROS1* rearrangement in NSCLC is estimated at 1–2%, and can be detected using FISH or IHC. Patients, similar to those with *ALK*-rearranged lung cancer, tend to be younger, never smokers, and have adenocarcinoma histology, although cases in squamous carcinoma have been reported (26). A response rate of 60% has been reported with crizotinib in 35 patients with *ROS-1*-rearranged lung cancer, including two patients with complete response, and median PFS was not reached (27). Multiple other agents are under development including AP26113, foretinib, PF-06463922, ceritinib, and HSP90 inhibitors such as AT13387 (NCT01712217).

### Mesenchymal–epidermal transition receptor

Mesenchymal–epidermal transition is a receptor tyrosine kinase, which undergoes homodimerization by binding its ligand, hepatocyte growth factor (HGF), to trigger intracellular signaling cascades, including PI3K–*AKT*–mTOR and RAS–RAF–MAPK pathways. In lung cancer, *MET* mutations are rare, but amplification is seen in up to 21%, resulting in constitutive *MET* activation and is believed to be a potential mechanism of acquired *EGFR* TKI resistance (28, 29). *MET* expression is seen in at least one-third of lung cancers, including adenocarcinoma and squamous histology (30).

Targeting *MET* protein-expressing lung cancer has not been successful to date, with negative phase III trials of onartuzumab (anti-*MET* monoclonal antibody), and TKIs including tivantinib (31, 32). Crizotinib activity has been reported in *MET*-amplified tumors (33), with ongoing studies in *EGFR* TKI-resistant lung cancer of *MET* and HGF-targeted agents, such as ficlatuzumab (anti-HGF monoclonal antibody, NCT02034981).

### *NTRK1* fusions

These have recently been described in never smokers with adenocarcinoma that is *ALK* and *EGFR* wild type. *NTRK-1* fusions have been identified in 3 of 91 lung adenocarcinoma samples that were *EGFR/KRAS/ALK-1/ROS-1* negative (34). RXDX101 has demonstrated activity in TRK-fusion positive lung cancer in a recent phase I trial (35).

## Targets within Intracellular Pathways

### Anaplastic lymphoma kinase

Anaplastic lymphoma kinase fusion genes, resulting in *ALK* fusion proteins, are present in 3–5% of lung adenocarcinomas, commonly in never smokers and younger patients. The presence of *ALK* fusion strongly predicts response to *ALK* TKIs, such as crizotinib, ceritinib, and others. This topic is discussed in length in a separate review article in this issue.

#### RAS–RAF–*MEK* pathway

The *RAS* family of oncogenes includes *H-RAS*, *K-RAS*, and *N-RAS*. RAS proteins encode a membrane-bound GTP-ase that mediates signal transduction from various tyrosine kinase receptors (e.g., *EGFR*, *HER2*) to the RAF/*MEK*/ERK pathway and others, regulating cell growth, proliferation, and apoptosis (36). *KRAS* mutations are seen in ~30% of Western adenocarcinoma cases, fewer in Asian populations, most commonly in codons 12 or 13. *NRAS* and *HRAS* mutations are less common in lung cancer, <1% (37).

### K-RAS

The role of mutant *KRAS* (V-Ki-ras2 Kirsten rat sarcoma viral oncogene homolog) as a prognostic or predictive marker in NSCLC remains controversial. An analysis of LACE-BIO suggests that it is not prognostic in early stage lung cancer, nor does it predict for adjuvant chemotherapy benefit (38). Several studies suggest that it is a potential negative predictor of benefit from *EGFR* TKI (39). While *KRAS* mutations have been identified in patients with and without smoking histories, never smokers are more likely to have transition mutations. Transversion mutations are found almost exclusively in smokers (40).

The most promising agents in development for *KRAS* mutant lung cancer have been *MEK* inhibitors combined with chemotherapy. Selumetinib, a MEK1/2 inhibitor, significantly improved PFS and response when added to docetaxel versus docetaxel plus placebo (HR = 0.58, *p* = 0.014, RR 37 vs. 0%, *p* < 0.0001), with a trend toward greater survival (41); a phase III trial is ongoing. Trametinib, another *MEK* inhibitor, showed activity in combination with docetaxel as well as with pemetrexed (42, 43). The response rate with single agent trametinib is 12%, with similar activity to docetaxel in pre-treated *KRAS* mutant lung cancer patients (44).

### *BRAF* 

*BRAF*, a serine-threonine kinase, lies downstream of *KRAS* and directly activates *MEK* by phosphorylation, which in turn activates ERK. *BRAF* (v-Raf murine sarcoma viral oncogene homolog B) mutations and *BRAF* inhibitors first gained attention in melanoma where 40–60% of tumors harbor activating V600E *BRAF* mutations. Three percent of lung adenocarcinomas harbor *BRAF* mutations, half of the V600E subtype, inducing constitutive kinase activity. These mutations occur more frequently in smokers. Dabrafenib, a *BRAF* kinase inhibitor, demonstrated a 54% RR in 17 *BRAF* V600E-mutated NSCLC patients (45). Vemurafenib is another *BRAF* kinase inhibitor that shown activity in this population. There are ongoing clinical trials assessing *BRAF*, *MEK*, and *AKT* inhibitors in this population.

#### PI3K–*AKT*–PTEN pathway

The phosphatidylinositol 3-kinase (PI3K)–*AKT*–mTOR (mammalian target of rapamycin) signaling pathway is one of the most dysregulated pathways in human cancers, including NSCLC. PI3K can be activated by transmembrane receptor tyrosine kinases like *EGFR* or RAS, through phosphorylation of *AKT*. This inhibits pro-apoptotic proteins and promotes cell survival. Activated mTOR complexes (mTORC1), downstream of PI3K–*AKT*, result in increased ribosomal protein synthesis and further *AKT* activation (mTORC2). PI3K-dependent signal transduction can be terminated by PTEN, a tumor suppressor intracellular protein (46).

### PIK3CA

*PIK3CA* encodes the catalytic subunit of PI3K, and mutations and amplification are seen in 2 and 12–17% of NSCLC cases (47, 48). These mutations can co-exist with other known driver mutations in lung adenocarcinoma, including *EGFR* and *KRAS* and in the setting of acquired *EGFR* TKI resistance (49, 50), suggesting that this may not be a driver mutation in itself. Trials of PI3K specific kinase inhibitors are ongoing.

### PTEN, *AKT*, mTOR

Loss of PTEN protein expression, with subsequent *AKT* overexpression, occurs in a third of NSCLC cases, and is associated with poor prognosis in lung cancer (51). This may be related to epigenetic silencing, as *PTEN* mutations are rare in NSCLC (52). *AKT* activation and mTOR phosphorylation is found in 51% of NSCLC cases, although *AKT* mutations are rare (<1%). Given the high level of activation and “crosstalk” with the RAS–RAF–*MEK* pathway, studies of mTOR and *AKT* inhibitors are of major interest in lung cancer. Everolimus (RAD001), temsirolimus, and other mTOR inhibitors are being investigated in combination with other targeted agents, including *EGFR* TKIs, although toxicity of these agents remains challenging, with high rates of fatigue and stomatitis (53, 54).

#### Wnt-beta-catenin pathway

The Wnt signaling pathway is highly active in lung cancer and correlates with metastasis and proliferation, and is believed to maintain cancer stem cells. Activated Wnt signaling inhibits the proteolysis of beta-catenin. Accumulated beta-catenin in cytoplasm moves to the nucleus where it initiates transcription factors promoting cell growth and chemo- and radio-resistance. Down-regulation of Wnt inhibitors is common in NSCLC samples and associated with poor prognosis (55). *WNT* mutations are rare in lung cancer and mutations in Beta-catenin are detected in 2% of lung adenocarcinoma (56). Several targeted therapies against the Wnt pathway are being investigated in early phase trials, including PRI-724, a small molecule beta-catenin inhibitor.

## Nuclear Targets

### Poly-ADP ribose polymerase

BRCA1, BRCA2, and PALB2 are proteins responsible for repair of DNA double-strand breaks through the homologous repair pathway; breaks that are not repaired lead to apoptosis. This repair pathway can be disrupted by mutations in *BRCA1*, *BRCA2*, or *ATM* (ataxia telangiectasia mutated), found in 7% of lung adenocarcinomas. High levels of BRCA1 protein expression in lung cancer correlate with poor survival, while decreased expression predicts response to platinum-based chemotherapy (57, 58). The poly-ADP ribose polymerase (PARP) enzyme is key in repairing single-strand DNA breaks, which may lead to double-strand breaks. BRCA deficient or mutated cells are sensitive to PARP inhibition, which may also sensitize cancer cells to alkylator or platinum damage via DNA single- or double-strand breaks. Despite a negative study with iniparib and chemotherapy, veliparib, and olaparib are being evaluated in combination with platinum-based therapy or *EGFR* TKI in NSCLC.

#### Heat shock protein 90

Heat shock protein 90 is a chaperone protein that assists posttranslational folding of several proteins to stabilize and protect them from cellular stresses like heat or hypoxia, including critical proteins in lung cancer such as *EGFR*, *HER2*, *MET*, *ALK*, and others. HSP90 inhibitors have shown activity in *EGFR* mutant lung cancer after the development of resistance, in *ALK*-rearranged tumors and more recently in *EGFR* wild type adenocarcinoma when combined with chemotherapy (59). A phase III clinical trial of docetaxel plus or minus ganetespib in chemo-naïve adenocarcinoma is ongoing. Other HSP90 inhibitors under active investigation in lung cancer include retaspimycin (IPI-504), AUY992, and AT13387.

#### Histone deacetylase

Histones are a family of proteins bound to DNA strands that maintain the helical structure of DNA. DNA expression is regulated by acetylation and deacetylation of histones. Deacetylation results in condensed DNA and reduced transcription. But histone deacetylase (HDAC), highly expressed in most cancers, may also alter activity of various proteins involved in carcinogenesis including HSP90, STAT3, and p53. HDAC inhibitors have multiple effects on DNA transcription, including induction of HSP90 acetylation (see above), disrupting its function, and resulting in tumor apoptosis. Vorinostat, FDA approved for treatment of cutaneous T-cell lymphoma, showed initial promise when added to chemotherapy in advanced NSCLC, although the subsequent phase III trial was negative (60). Other HDAC inhibitors being studied include etinostat, romidepsin, pabinostat, pivanex, and CI-994.

## Targets in the Tumor Environment

### Angiogenesis

Vascular endothelial growth factor (VEGF) is a pro-angiogenic factor, with a key role in tumor angiogenesis. Its high expression in a variety of tumors, including NSCLC, is associated with poor prognosis (61). Although multiple agents targeting VEGF and VEGF receptors have been studied in lung cancer, only bevacizumab and more recently nintedanib have improved survival in advanced non-squamous NSCLC. Bevacizumab, combined with paclitaxel and carboplatin, improved response, PFS, and survival in the practice-changing ECOG4599 trial (62), although subsequent bevacizumab trials have not improved survival compared to chemotherapy alone. Nintedanib, a multi-targeted VEGF- and FGFR-1 receptor TKI demonstrated greater OS in a subgroup of adenocarcinoma patients when added to docetaxel versus chemotherapy alone (63). Trials of multiple other agents have not demonstrated positive results, although trials with VEGF/R inhibitors, including in different molecular subtype of adenocarcinoma, are ongoing.

Vascular disrupting agents, such as vadimezan, target vasculature directly, not through VEGF/VEGFR. To date, trials of these and multiple other anti-angiogenic agents have not yet yielded benefit.

### Immune modulation

The immune system plays an active role in eradication of malignant cells. However, the evolution of cancer includes developing mechanisms to escape the immune system. Several approaches are now being investigated to boost anti-cancer immune response, either by inhibiting immune checkpoints (as CTLA-4, PD-1, and PD-L1) or by developing vaccines of cancer antigens. This topic is discussed in length in a separate review article, with the PD-1 checkpoint inhibitors as the most promising current target in immune therapy of lung cancer, with demonstrated single agent activity in both adenocarcinoma and squamous carcinoma (64).

There are multiple other potential targets in lung adenocarcinoma that are not reviewed here, such as the cell surface receptor insulin-like growth factor 1 receptor, apoptotic receptors, and proteins including TRAIL, BCL-1, IAP proteins including survivin, and the proteasome. Additional targets in the tumor environment include adhesion molecules such as integrins, and even osteoclasts, all potentially important targets in lung cancer with ongoing trials of targeted agents.

## Conclusion

Striking therapeutic advances in metastatic NSCLC have been observed with targeted agents using molecular selection, notable for patients with *EGFR* mutant or *ALK*-rearranged lung cancer. Testing for these oncogenic drivers is now standard of care in advanced lung adenocarcinoma, but they are found in only ~20% of lung adenocarcinomas in Western populations, while remaining patients are eligible only for standard chemotherapy. However, this “success story,” as well as improved understanding of molecular pathways of lung carcinogenesis, had led to rapid progress in the identification of novel targets in adenocarcinoma and potential therapies. Despite this enthusiasm, there are still barriers to overcome, including how to approach tumors without single oncogene addiction, i.e., targeting multiple pathways, and also how to overcome primary and secondary resistance to targeted therapies. Finally, the development of accurate, rapid, tissue-, and cost-conserving assays to identify multiple targets simultaneously, including targets beyond genomic sequencing, is urgently needed. In the meantime, drug development and discovery of novel targets in lung adenocarcinoma remain one of the fastest growing areas of research and development in oncology today.

## Conflict of Interest Statement

The authors declare that the research was conducted in the absence of any commercial or financial relationships that could be construed as a potential conflict of interest.
